# Gestational and congenital syphilis: gaps to be elucidated

**DOI:** 10.1590/0037-8682-0038-2023

**Published:** 2023-06-02

**Authors:** Déborah Esteves Carvalho, João Victor Andrade Pimentel, Luise Oliveira Ribeiro da Silva, Letícia Maria Cardoso Lima Rodrigues, Leonardo Santana Andrade, Carlos Ramon Costa Santana, Marcelo Antônio Silva Menezes, Eloyse Emanuelle Nunes Silva, Gabryelle Eduarda Gama dos Santos, Sayron Natanael Lopes Pereira Santos, Breno Gustavo do Nascimento Gomes, Letícia Almeida Meira, Helga Machado de Farias Santos, Izailza Matos Dantas Lopes

**Affiliations:** 1 Universidade Tiradentes, Departamento de Medicina, Aracaju, SE, Brasil.; 2 Universidade Federal de Sergipe, Departamento de Medicina, São Cristóvão, SE, Brasil.

**Keywords:** Gestational syphilis, Treponemal tests, Early congenital syphilis

## Abstract

Congenital and gestational syphilis are increasingly prevalent multisystemic infections in Brazil. This study aimed to present a case series of three children diagnosed with congenital syphilis even though their mother had unreactive treponemal tests. The VDRL (Venereal Disease Research Laboratory) titers of a 22-year-old mother with three pregnancies decreased after treatment. The mother did not have a reactive treponemal test, but all the three children were diagnosed with early congenital syphilis. This case series highlights the difficulty in diagnosing gestational and congenital syphilis in Brazil.

## INTRODUCTION

Gestational syphilis (GS) is a worldwide public health problem that causes social, economic, and health-related damage. The severity of sexually transmitted infections varies significantly, with congenital syphilis (CS) representing the worst possible outcome. Approximately two million pregnant women are estimated to present with the active form of the infection yearly, and < 10% are diagnosed and treated^1^. Several maternal risk factors, including inadequate prenatal care, low socioeconomic status, HIV (Human Immunodeficiency Virus) co-infection, drug use, teenage pregnancy, risky sexual behavior, and non-treatment of the infected partner, are associated with a higher incidence of GS^2^.

Immunological assays (nontreponemal and treponemal) that detect antibodies in whole blood, serum, or plasma samples are primarily used to diagnose syphilis. 

Non-treponemal tests detect anticardiolipin antibodies (IgM and IgG) by sample dilution. As these antibodies can be produced in conditions other than syphilis, such as systemic lupus erythematosus and leprosy, their limited sensitivity can lead to false-positive results. In Brazil’s public health system, the VDRL (Venereal Disease Research Laboratory) test is the non-treponemal test widely used to investigate active syphilis and monitor treatment through titration at diagnosis and post treatment^3^ ^,^ ^4^ ^.^

For the diagnosis of CS, when the pregnant woman is adequately treated, it is necessary that the result of non-treponemal tests of the newborn be higher than that of the mother in at least two dilutions. Lower dilutions occur because of the possibility of passing maternal IgG antibodies to the baby during pregnancy^5^ ^,^ ^6^. 

No cases series have been described in the Brazilian literature, in databases such as PubMed Central, PubMed, and SciELO, in which children with typical alterations of CS had unreactive treponemal test (FTA-ABS, Fluorescent treponemal antibody absorption, and the rapid test), reactive non-treponemal tests, with high titers. On receiving appropriate treatment, they showed reduced VDRL titer and unreactive result.

 This study aimed to present a case series in which three infants were diagnosed with early CS according to the Brazil’s Ministry of Health criteria^7^. However, the mother had non-reactive treponemal tests during all pregnancies, which, according to the current literature, would rule out the diagnosis of acquired or GS.

## CASE REPORT

**Mom:** A 22-year-old G3P3A0 secondary school-graduated housewife, from the city of Aracaju Sergipe, an urban area, had her first child at the age of 16. She was diagnosed with GS, and was administered penicillin G benzathine (2.4 million IU) in three doses at 7-day intervals. Additionally she has an untreated partner with a non-reactive VDRL result.

**During the first pregnancy:** She attended three prenatal consultations, starting from the 12th week of pregnancy. She presented with a VDRL titer of 1:16 at 6 months of gestation and was treated with three doses of benzathine penicillin (2.4 million IU). At 3 months postpartum, she presented with a VDRL titer of 1:16, and because her partner was untreated, the treatment was repeated. She presented with a VDRL titer of 1:16 1 year and 6 months postpartum and was treated again. The FTA-ABS treponemal test was conducted, with non-reactive IgG and IgM results, despite the fact that she had previously presented with the disease, which was diagnosed using non-treponemal tests.

**During the second pregnancy:** Her second child was female, born at 35 weeks (premature delivery) in December 2018 from her second relationship and current steady partner. During the pregnancy, the VDRL titer was 1:16, and the patient was administered six doses of benzathine penicillin G (two doses of 1.2 million IU given thrice) at 7-day intervals.

**During the third pregnancy:** Her third child was born at term by cesarean section. She attended nine prenatal consultations starting from the first trimester. After giving birth, she underwent definitive sterilization via tubal ligation. She underwent a rapid treponemal test in the first trimester, which was unreactive. However, non-treponemal tests during pregnancy and childbirth remained reactive (Table 1), and she was administered the same treatment regimen in the postpartum period. Her steady partner remained unreactive. She was referred to an infectious disease specialist, who conducted tests to exclude illnesses in which a non-treponemal test might yield a reactive result due to cross-reactivity.

**TABLE 1:** Correlation between values of performed tests, pregnancy, and period (mother).

Periods	First pregnancy	Second pregnancy	Third pregnancy
**First trimester**	not reported	not reported	VDRL 1/4
			FTA-ABS IgG and IgM non-reactive
**Third quarter**	VDRL 1/16	VDRL 1/16	VDRL 1/2
	(pre-treatment titration, when benzathine penicillin 2.4 million IU was started with three doses and 7-day interval)	FTA-AB IgG and IgM non-reactive	(Others not performed)
		(pre-treatment titration, when six doses of benzathine penicillin 1.2 million IU were started with a 7-day interval)	
**One month before delivery**	not reported	VDRL 1/2	not reported
		(post-treatment titration)	
**At childbirth**	VDRL 1/8 (post treatment titration)	VDRL 1/2	VDRL 1/8
			Negative rapid test
			(pre-treatment titration, when six doses of benzathine penicillin 1.2 million IU were started with a 7-day interval)
**Three months postpartum**	VDRL 1/16	not reported	VDRL 1/2
			(post treatment titration)
**One year after giving birth**	non-reactive VDRL	VDRL 1/32 (Others not performed)	not reported
	FTA-ABS IgG and IgM non-reactive		
**One year and 3 months after giving birth**	VDRL 1/32.	not reported	not reported
	FTA-ABS IgG and IgM non-reactive		

**VDRL:** Venereal Disease Research Laboratory; **FTA-ABS:** (Fluorescent treponemal antibody absorption.

**FIGURE 1:**
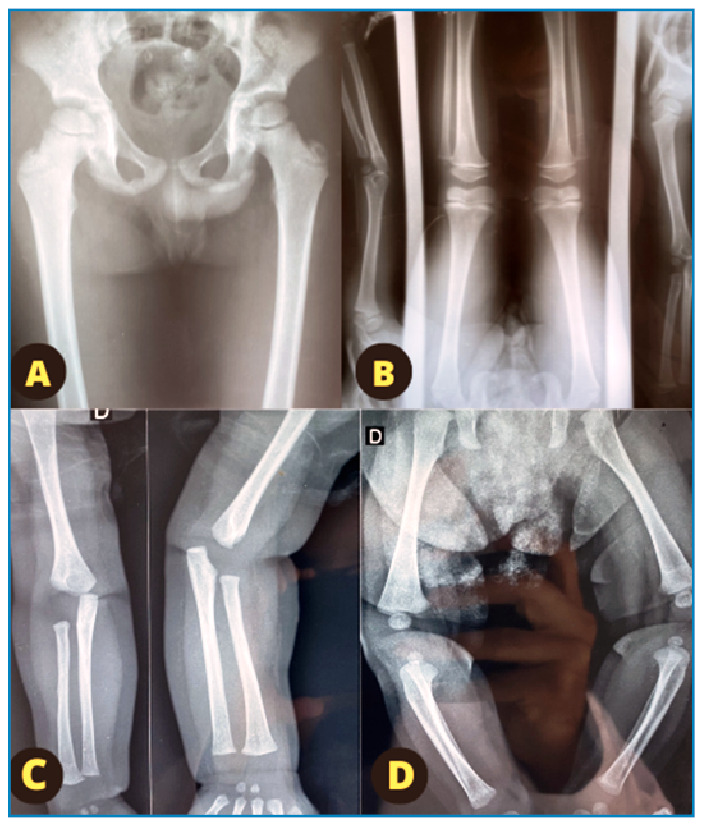
In **A:** leg radiograph showing no alterations in the first child after epiphyseal osteosclerosis in long bones from previous years. **In B:** diffuse metaphyseal alterations, suggestive of syphilis, are observed in the second child at the age of 2 years and 8 months. **In C and D:** metaphyseal alterations in the long bones of the third child (2 months old) are observed.

**First child**: A boy, with APGAR scores of 8 and 9 in the 1st and 5th min, respectively. born at term by cesarean section, with a VDRL titer of 1:2 and unreactive CSF (Cerebrospinal Fluid) analysis. He was treated with procaine penicillin for 10 days after birth. Follow-up radiography of (Figure 1A), revealed radiotransparency alterations in all of his long bones, suggestive of syphilis. CSF analysis and fundoscopy revealed no alterations; however, he failed the auditory test, with a low bilateral response (at 3 months, the BERA, Brainstem Evoked Response Audiometry, was normal). He maintained adequate growth throughout the follow-up period (between +2 and -2 scores for height and weight). Regarding neuropsychomotor development, speech delay was noticed at the age of 2, persistent language delay at the age of 3, and aggression was reported at the age of 5 (suspected behavioral disorder).

**Second daughter**: A female, born in December 2018, with different parent. She was admitted to the neonatal intensive care unit immediately after birth with bronchopneumonia, jaundice, anemia, and hypocalcemia. She was administered penicillin for 7 days, ceftriaxone for 3 days, and ampicillin and gentamicin for 7 days to treat CS. The VDRL test and CSF analysis were non-reactive at birth. Fundoscopy and auditory examinations were normal, and the NPMD (Neuropsychomotor Development) was adequate in all consultations (between +2 and -2 scores for height and weight). At the ages of 3 and 6 months, she presented with non-reactive VDRL. At birth, metaphyseal changes were detected on radiographs of all long bones. At the ages of 1 and 2 years, she continued to exhibit metaphyseal changes in all long bones (Figure 1B) and non-reactive VDRL. 

Third child: Third child, a female, born in 2022 by cesarean section at term, with APGAR scores of 9 and 10 in the 1st and 5th min, respectively. The mother attended nine prenatal consultations starting from the first trimester of the pregnancy. At birth, the VDRL titer was 1:4 and the auditory and fundoscopy test results were normal. Thus, according to the CS definition criteria updated in 2017, she was exposed to *Treponema pallidum* but was not infected. Bone radiograph performed at 2 months of age revealed metaphyseal alterations in all long bones (Figure 1C-D). These alterations confirmed the diagnosis of CS, which was treated with crystalline penicillin for 10 days; at 3 months, the VDRL test was unreactive. During childcare consultations, at 2, 3, and 7 months, she showed adequate growth, always between +2 and -2 Z-scores, and adequate neuropsychomotor development.

## DISCUSSION

In recent years, doctors have had difficulty diagnosing syphilis because of the pathogen’s characteristics, including a few trackable proteins in its outer membrane^8^ ^,^ ^9^. Direct detection tests (dark field microscopy and polymerase chain reaction) enable the identification of *T. pallidum*; however, they have a limitation that prevent their widespread use in clinical practice^8^, such as their inapplicability in asymptomatic patients who constitute the majority of the infected individuals^7^.

Despite their low sensitivity^9^, non-treponemal tests are essential for diagnosing active syphilis and assessing the efficacy of treatment^10^. Although most immunocompetent patients show non-reactive results between 6 months and 2 years of successful treatment, up to 20% of infected patients show persistently reactive results at low titers (usually below 1:8), known as immunological scarring^,^ ^11^ ^,^ ^12^.

Routinely, a nontreponemal test is followed by a treponemal confirmatory test^8^ ^,^ ^12^. However, reverse algorithms, in which the treponemal antibody test is performed first, followed by a non-treponemal test,^8^ are recommended by the Brazilian Ministry of Health as a diagnostic protocol^7^. 

Despite the screening, diagnosis, follow-up, and treatment strategies for GS, CS occurred in this case. Available serological tests are effective, inexpensive, and allow for longitudinal follow-up of the disease. However, they have significant false-positive and false-negative rates. Therefore, additional strategies are required to manage this disease better.

This case series highlights the apparent discrepancies in the diagnostic evaluation of a mother with syphilis whose treponemal tests (FTA-ABS and rapid test) were unreactive with reactive non-treponemal tests with high titers. Following treatment with an appropriate dose and interval of benzylpenicillin, VDRL titers decreased. The metaphyseal changes in the long bones of all three children indicated CS, as evaluated by two different radiologists who confirmed the diagnosis (Table 2).

**TABLE 2:** Chronological correlation between the long bone radiographic findings of the three children.

	First child	Second child	Third child
**At birth**	Radiodensity changes in all long bones	Metaphyseal changes in all long bones	Metaphyseal changes in all long bones
**6 months**	Epiphyseal osteosclerosis in all long bones		
**12 months**		Metaphyseal changes in all long bones	
**24 months**	Metaphyseal osteosclerosis in all long bones		
**36 months**	Metaphyseal osteosclerosis in all long bones	Metaphyseal changes in all long bones	
**60 months**	Without changes		

A 2017 article published in the Brazilian Journal of Orthopedics described a case of a newborn with CS with a typical bone lesion who showed reactive VDRL result at birth, and a mother with reactive VDRL result during pregnancy (VDRL 1:32), which was untreated. Another article published in 2020 by the Brazilian Journal of Pediatrics described a case of a newborn with palmoplantar pemphigus lesions who was screened for CS; a blood VDRL of 1:512 in the newborn and 1:128 in the mother during postpartum period confirmed the diagnosis of CS. 

In conclusion, this case series highlights the importance of investigating CS in children with typical radiological findings, even if treponemal tests are unreactive and non-treponemal tests with high titers are reactive. Clinicians should be aware of the possibility of false-negative treponemal tests because of early infection, previous treatment, or immunosuppression and rely on non-treponemal tests and clinical evaluation to guide the diagnosis and management of CS.
